# Effect of Cr Content on Microstructure and Mechanical Properties of Heat Affected Zone in Supercritical Carbon Dioxide Transport Pipeline Steel

**DOI:** 10.3390/ma18112607

**Published:** 2025-06-03

**Authors:** Rui Hong, Xiaodan Zhu, Shubiao Yin, Nengsheng Liu, Shujun Jia, Yuxi Cao, Yuqin Qin, Qilin Ma

**Affiliations:** 1 Faculty of Metallurgical and Energy Engineering, Kunming University of Science and Technology, Kunming 650093, China; 2 Department of Structural Steels, Central Iron and Steel Research Institute, Beijing 100081, China; 3 China Petroleum Pipeline Engineering Corporation, Langfang 065000, China; zhuxd@pipechina.com.cn

**Keywords:** pipeline steel, Cr element, supercritical CO_2_ transportation, M-A constituent, low temperature toughness

## Abstract

This study systematically investigates the influence mechanism of the element Cr on the mechanical properties of the heat-affected zone in pipeline steels for supercritical CO_2_ transportation. Microstructural evolution in the heat affected-zone was characterized through thermal simulation tests, Charpy impact testing (−10 °C), and microhardness measurements, complemented by multiscale microscopic analyses (optical microscopy, scanning electron microscopy, electron backscatter diffraction, and transmission electron microscopy). The results demonstrate that Cr addition enhances the base metal’s resistance to supercritical CO_2_ corrosion but reduces its low-temperature impact toughness from 277 J to 235 J at −10 °C. Notably, the intercritical heat-affected zone exhibits severe embrittlement, with impact energy plummeting from 235 J (base metal) to 77 J. Microstructural analysis reveals that Cr interacts with carbon to form stable carbonitride particles, which reduce the free carbon concentration and diffusion coefficient in austenite, thereby inducing heterogeneous austenitization. Undissolved carbonitrides pin grain boundaries, creating carbon concentration gradients. During rapid cooling, these localized carbon-enriched microregions preferentially transform into core–shell-structured M-A constituent, characterized by a micro-twin containing retained austenite core encapsulated by high hardness lath martensite. The synergistic interaction between micro-twins and interfacial thermal mismatch stress induces localized stress concentration, triggering microcrack nucleation and subsequent toughness degradation.

## 1. Introduction

With the rapid development of global carbon capture and storage (CCUS) technology, supercritical carbon dioxide (S-CO_2_) delivery pipelines, as critical infrastructure, are facing severe operational environmental challenges [1,2]. Although the high density and low viscosity characteristics exhibited by S-CO_2_ above the critical point (31.1 °C, 7.38 MPa) are conducive to improving the transportation efficiency, the coupling effect of its strong permeability and acidic impurities (such as H_2_S, SO_2_, NOx, etc.) is prone to causing localized corrosion and hydrogen induced cracking of pipeline steel [3,4,5,6]. Therefore, in the past few years, the corrosion problem of supercritical carbon dioxide transportation pipelines containing impurities has been a research hotspot [7,8,9,10,11,12]. To address the potential impacts of corrosion, the corrosion resistance of steel is generally improved by adding corrosion resistant elements such as Cr. However, the addition of Cr significantly affects the weldability of steel. Specifically, the combined effects of thermal conduction and high heat input during welding lead to the formation of residual stresses in the welding heat-affected zone (HAZ). As the weakest link in pipeline structures, the stress distribution within the HAZ during long term service is prone to induce brittle fracture and stress corrosion failure, posing a potential threat to the safety of pipeline transportation systems. Therefore, achieving both enhanced corrosion resistance and favorable weldability is critically important [13,14,15].

Traditional X65 pipeline steel exhibits significant corrosion rates in water-saturated S-CO_2_ environments, failing to meet the corrosion resistance requirements for materials in high temperature and high-pressure S-CO_2_ transportation systems [16]. Micro-alloyed composition design is a critical approach to enhancing the comprehensive performance of pipeline steel. Among alloying elements, Cr has been widely studied for its corrosion resistance benefits. Wang Bei investigated the influence of varying Cr content on steel’s corrosion resistance, concluding that the corrosion resistance of 1 Cr–6.5 Cr steel correlates with its pseudo passivation capability. This phenomenon arises from the formation of a Cr(OH)_3_ based corrosion product film on the steel surface [17]. Qiao Shuang’s comparative study on the corrosion resistance of various metallic materials revealed that austenitic stainless steels and nickel-based alloys exhibit superior resistance to supercritical carbon dioxide compared to ferritic–martensitic steels. This enhanced performance can be attributed to their higher Cr content and other alloying elements, which facilitate the formation of a dense protective oxide layer that effectively inhibits further corrosion progression [18]. Cai-Lin Wang et al. revealed that the general and localized corrosion behavior of materials is closely tied to the formation and evolution of corrosion products. Cr addition suppresses both general and localized corrosion in early stages. However, as corrosion products evolve, dense crystalline FeCO_3_ layers form on X65 and 1Cr steel surfaces, significantly lowering general corrosion rates [19]. Research on Cr content’s impact on pipeline steel weldability has primarily focused on welding materials or weld metal, with limited studies on the heat affected zone (HAZ) [20,21,22,23,24,25,26]. Wang Feng et al. explored factors influencing unstable impact toughness in self-shielded flux-cored wire welds, noting that Cr increases weld hardenability, delays pearlite transformation, and promotes bainite formation [24]. Hou Yu demonstrated that Cr enhances bainitic transformation kinetics, expanding the granular bainite formation range during cooling. This results in increased granular bainite content, larger martensite–austenite (M-A) constituent sizes, and reduced weld impact toughness [25]. Wang Yuefeng found that higher Cr content under fixed welding heat input increases lath bainite (LB) content, decreases granular bainite (GB) content, raises M-A constituent volume, reduces high angle grain boundary (HAGB) proportions, and elevates hardness in the coarse-grained heat affected zone (CGHAZ) [26]. However, the influence of Cr content in supercritical CO_2_ transportation pipeline steel on HAZ performance remains underexplored. This study investigates X65 grade pipeline steel with controlled Cr content (0.2~0.5 wt.%) to simulate welding HAZ thermal cycles using a Gleeble 3800 thermomechanical simulator. Microstructural evolution in different HAZ subzones is characterized by SEM, EBSD, and TEM, while fracture toughness variations are evaluated through impact testing. The systematic analysis of Cr’s effects on HAZ microstructure and mechanical properties aims to provide theoretical guidance for optimizing the composition of next-generation supercritical CO_2_ pipeline steels.

## 2. Experimental Materials and Methods

### 2.1. Experimental Materials

The experimental materials employed in this study consist of commercial X65 pipeline steel and laboratory-prepared steel. The laboratory produced steel was processed into rolled plates through a controlled rolling and cooling (TMCP) technique. For convenience of reference, the commercial X65 pipeline steel will be designated as Steel A, while the laboratory-produced steel will be referred to as Steel B throughout this paper. The chemical compositions of these steels, determined in accordance with the Chinese standard GB/T 4336-2016 [27] using spark discharge optical emission spectrometry (OES), are presented in Table 1. The primary distinction between these materials lies in their Cr content variations. In the evaluation system for low-alloy steels, the carbon equivalent (*CE*_(IIW)_) serves as a critical quantitative indicator, with its numerical value directly correlated to the material’s weldability performance. This parameter typically exhibits a negative correlation characteristic, where a lower CE_IIW value indicates a reduced risk of cold cracking during welding processes and enhanced adaptability to welding techniques. The calculation formula is expressed as Equation (1) [28]:

(1) $${\;CE}_{\left(\mathrm{II}W\right)}=w(C)+\frac{w(Mn)}{6}+\frac{w(Cr+Mo+V)}{5}+\frac{w(Cu+Ni)}{15}$$

**Figure 1:**
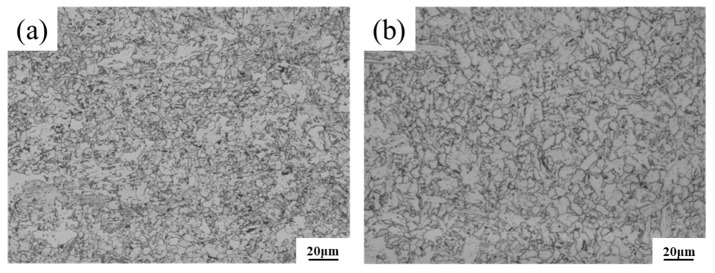
Metallographic structure of experimental steel base metal: (**a**) Steel A; (**b**) Steel B.

**Figure 2:**
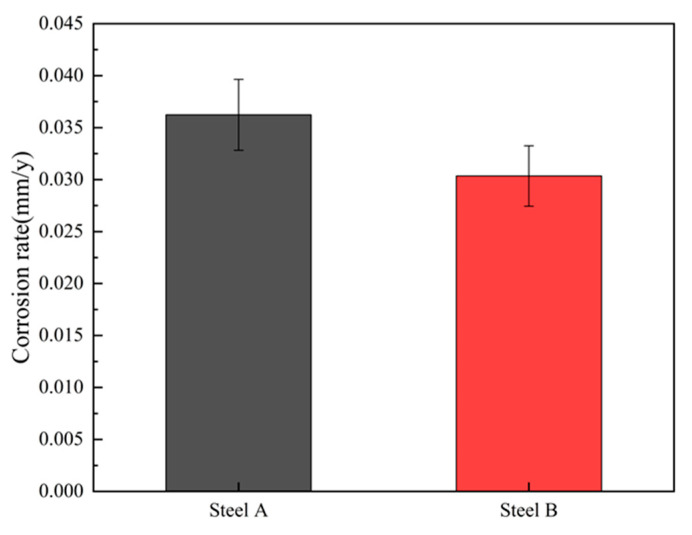
Corrosion rate of experimental steel.

The calculated carbon-equivalent (*CE*_(IIW)_) values for Steel A and Steel B are 0.0394 and 0.454, respectively. Notably, while the *CE*_(IIW)_ of Steel B exhibits a slight increase, it remains within a low threshold, ensuring that both experimental steels can be processed using the identical welding simulation parameters. Figure 1 presents the microstructure of Steel A and Steel B. The microstructure of the two steels is mainly a combination of granular bainite and polygonal ferrite, ensuring a good match of strength and toughness for X65 pipeline steel. Figure 2 presents the corrosion rates of the two steels in supercritical CO_2_ environments. The test steels were machined into corrosion coupons with dimensions of 50 mm × 10 mm× 2.7 mm. Triplicate specimens were prepared for each condition. The experiments were conducted in a 3 L autoclave under water-saturated supercritical CO_2_ conditions (containing 10 g H_2_O) at 10 MPa and 50 °C. After 168 h of exposure, the specimens were retrieved, dried, and pickled to obtain corrosion data. Steel B, with higher Cr content, exhibited an increased proportion of granular bainite and demonstrated enhanced corrosion resistance.

### 2.2. Welding Thermal Cycle Experiment

**Figure 3:**
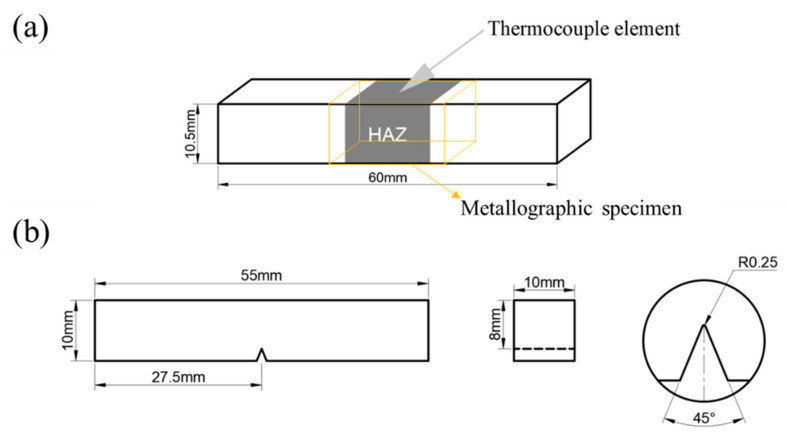
Sample processing diagram: (**a**) thermal simulation sample and metallographic sample; (**b**) impact sample.

**Figure 4:**
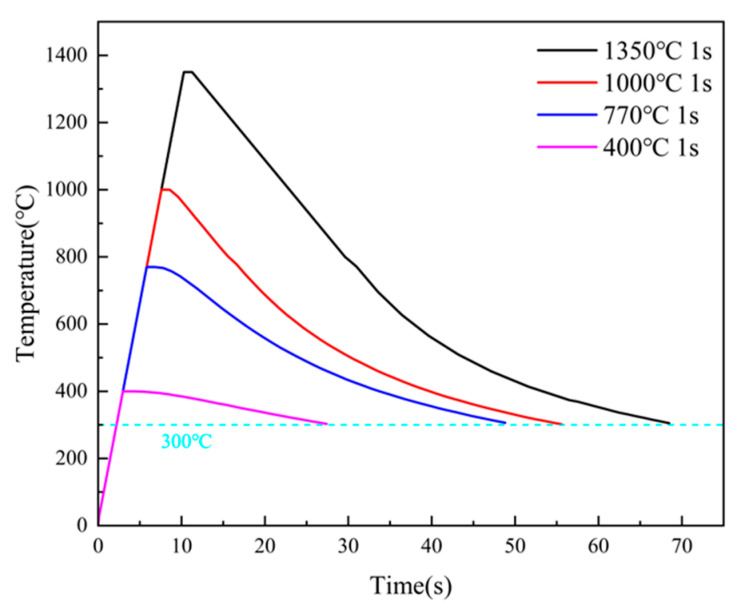
Thermal simulation cycle curves for different peak temperatures.

The phase transformation critical points Ac₁ and Ac₃ of the experimental steels were determined using the tangent method based on temperature dilation curves. The values for Steel A were 680 °C/890 °C, and for Steel B, 688 °C/910 °C. Specimens of 10.5 mm × 10.5 mm × 60 mm were cut along the rolling direction of the laboratory-rolled plates and subjected to thermal simulation experiments using a Gleeble 3800 thermal mechanical simulator, as shown in Figure 3a. The experiment utilized a welding heat input of 30 kJ/cm to simulate the single-pass welding process of X65 pipeline steel under actual production conditions. Based on the phase transformation critical points of the experimental steels, different peak temperature intervals were set: CGHAZ: 1350 °C; fine-grained heat-affected zone (FGHAZ): 1000 °C; intercritical heat-affected zone (ICHAZ): 770 °C; subcritical heat-affected zone (SCHAZ): 400 °C. The thermal simulation cycle curves for different peak temperatures are illustrated in Figure 4.

### 2.3. Microstructure and Fracture Surface Observation and Analysis

For microhardness testing, specimens (20 mm × 10 mm × 10 mm) were ground and polished, followed by five-point Vickers hardness measurements at the central region using an MH-500D semi-automatic micro-Vickers hardness tester under a 500 g load. Extreme values were excluded, and the average of three valid measurements was recorded, as illustrated in Figure 3a. Metallographic specimens with identical dimensions were prepared and observed under an MEF-4M metallographic microscope to examine microstructural features across multiple fields of view.

Thermally simulated specimens were machined into standard Charpy V-notch impact specimens (10 mm × 10 mm × 55 mm, Figure 3b) and tested at −10 °C in accordance with the GB/T 229-2020 standard [29]. The average impact absorbed energy of three parallel specimens per parameter set was adopted as the characteristic value.

For electron backscatter diffraction (EBSD) analysis, specimens were electrolytically polished using a 6% perchloric acid–alcohol solution (pre-cooled) at 20 V for 15 s with an Electromet 4 electrolytic polisher to eliminate surface stress. EBSD scans were performed on the heat affected zone using a ZEISS Sigma 500 field-emission scanning electron microscope (FE-SEM) with an optimized step size of 0.4 μm.

Transmission electron microscopy (TEM) samples were prepared by wire-cutting 0.3 mm slices, mechanically grinding to 50 μm thickness, and punching into 3 mm discs. Final thinning was achieved via twin-jet electropolishing in a liquid nitrogen environment (−20~−30 °C). Microstructural characterization was conducted using a Talos F200X G2 TEM.

## 3. Experimental Results and Analysis

### 3.1. Microstructure Analysis

**Figure 5:**
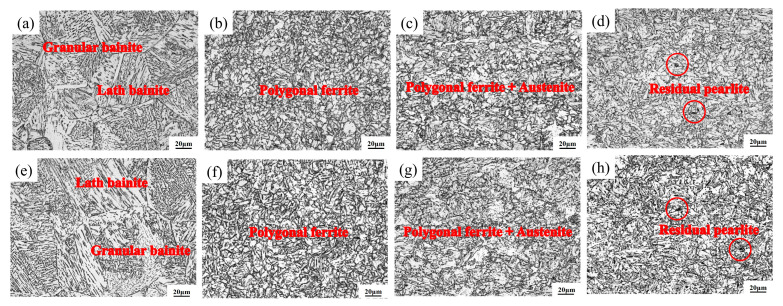
Metallographic structure of different HAZ subregions of experimental steel: (**a**) Steel A 1350 °C; (**b**) Steel A 1000 °C; (**c**) Steel A 770 °C; (**d**) Steel A 400 °C; (**e**) Steel B 1350 °C; (**f**) Steel B 1000 °C; (**g**) Steel B 770 °C; (**h**) Steel B 400 °C.

**Figure 6:**
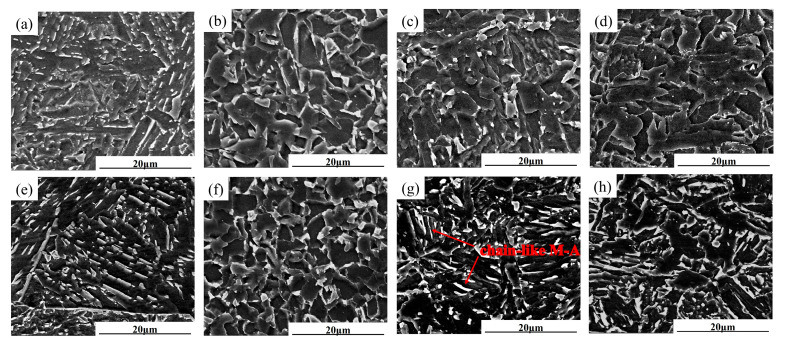
SEM microstructure characterization of different HAZ subregions of experimental steel. (**a**) Steel A 1350 °C; (**b**) Steel A 1000 °C; (**c**) Steel A 770 °C; (**d**) Steel A 400 °C; (**e**) Steel B 1350 °C; (**f**) Steel B 1000 °C; (**g**) Steel B 770 °C; (**h**) Steel B 400 °C.

Figure 5 and Figure 6 show the microstructure of different subzones in the HAZ of the experimental steel under a heat input of 30 kJ/cm. As observed, the HAZ microstructures of all three experimental steels exhibit distinct gradient characteristics under controlled peak temperature conditions. When the peak temperature range significantly exceeds Ac_3_ (1350~1100 °C), this corresponds to the CGHAZ. Under extremely high thermal exposure, the material undergoes complete austenitization, leading to the full dissolution of carbonitride particles originally precipitated within the parent austenite matrix. The absence of these particles removes their “pinning effect” on grain boundaries, leading to rapid austenite grain growth (expanding from the original 10~20 μm in the base metal to 80~120 μm). In the temperature range of 1100 °C to Ac_3_, the microstructure undergoes complete phase transformation and recrystallization during heating, forming fully austenitized structures. Subsequent cooling drives further structural reorganization through phase decomposition. These consecutive transformations refine the microstructure significantly. Coupled with relatively low heat input, this results in incomplete austenite coarsening, reduced bainitic lath morphology, and ultimately a mixed structure of polygonal ferrite and granular bainite with grain sizes of 5–10 μm. In the ICHAZ, unrecrystallized coarse original ferrite grains coexist with newly formed austenite. Notably, Steel B exhibits chain-like M-A constituent at grain boundaries (Figure 6g). Below Ac_1_, no recrystallization occurs, but a brief tempering process takes place. Original pearlite undergoes carbide spheroidization, where cementite particles transition from lamellar to spherical morphology with reduced volume fraction. This produces a microstructure dominated by granular bainite, polygonal ferrite, and minor residual pearlite.

### 3.2. Mechanical Property Analysis

**Figure 7:**
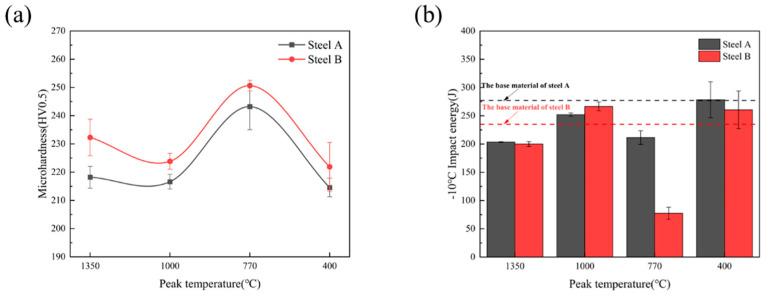
Influence of different peak temperature on mechanical properties of experimental steel (**a**) microhardness; (**b**) impact toughness.

Figure 7a shows the curve of microhardness versus peak temperature for the experimental steel. As clearly observed from the figure, the overall trend of microhardness variation in the experimental steel remains consistent across different peak temperatures. Specifically, no significant changes occurred in the CGHAZ and FGHAZ, where the microhardness of the experimental steel showed only a slight increase compared to the base material, though not pronounced. However, upon entering the CHAZ, the microhardness of the experimental steel increased markedly, reaching peak values of 243 HV0.5 and 253 HV0.5 at 770 °C, with both values exceeding the base material by approximately 30 HV0.5. Subsequently, in the subcritical HAZ (SCHAZ, 400 °C), the microhardness began to decline, gradually decreasing to match that of the base material as the peak temperature further decreased. Notably, under identical peak temperatures, the microhardness of the experimental steel increased with higher Cr content.

Figure 7b shows the curve of impact toughness of the experimental steel as a function of peak temperature. As can be seen from the figure, with decreasing peak temperature, the impact energy generally exhibits an initial increase followed by a decrease and then another increase. At a peak temperature of 1350 °C, the impact energy in this region remained above 150 J, indicating no significant deterioration in low temperature toughness. When the peak temperature decreased to 1000 °C, the refined microstructure endowed this region with excellent low temperature toughness, with impact energy values around 250 J. However, as the temperature further dropped to 770 °C, the low temperature toughness of Steel B deteriorated markedly, showing an impact energy of only 77 J—representing over 67% loss compared to the base metal. This degradation is attributed to microstructural characteristics (type, proportion, and size) influenced by incomplete austenitization. When the peak temperature fell below 700 °C, the impact energy recovered to levels comparable with the base metal, as the microstructure differed minimally from the parent material except for undergoing brief tempering.

### 3.3. Analysis of Fracture Surface Morphology

**Figure 8:**
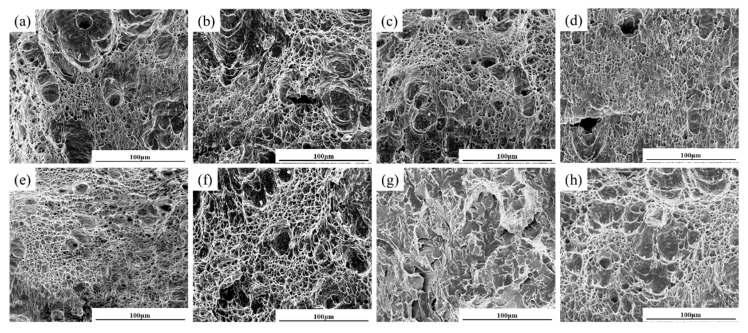
Impact fracture of experimental steel at different peak temperatures: (**a**) Steel A 1350 °C; (**b**) Steel A 1000 °C; (**c**) Steel A 770 °C; (**d**) Steel A 400 °C; (**e**) Steel B 1350 °C; (**f**) Steel B 1000 °C; (**g**) Steel B 770 °C; (**h**) Steel B 400 °C.

Figure 8 shows the impact fracture morphology of the experimental steel. It can be observed that after undergoing welding thermal cycles with various peak temperatures, different subregions in the weld heat affected zone exhibit distinct characteristics on their fracture surfaces. When the peak temperature reached 1350 °C, the fracture surface in this region was predominantly characterized by prominent dimple structures. As the temperature decreased to 1000 °C, the fracture morphology remained dominated by dimples, with relatively large numbers of deep, equiaxed dimples observed. This phenomenon is attributed to the finer grain structure in this region, where effective obstruction of crack propagation by grain boundaries increases the difficulty of crack advancement, thereby endowing this zone with excellent low-temperature toughness. For Steel A at 770 °C, while dimple structures still predominated, the dimples appeared larger and sparser. The uniform distribution of fine ferrite and bainite structures within the material served as the primary factor promoting dimple formation [30]. Additionally, the homogeneous dispersion of M-A constituent further alleviated excessive stress concentration at particles, allowing for this region to maintain good low-temperature resistance [31]. In contrast, Steel B at the same 770 °C peak temperature exhibited quasi-cleavage fracture as the dominant morphology in the fracture surface, with localized areas showing mixed dense dimple structures. The presence of carbide particles at grain boundaries likely acted as crack initiation sources, facilitating the development of cleavage crack directions and consequently leading to reduced low temperature toughness [32]. When the peak temperature reached 400 °C, the fracture morphology displayed significant characteristics of coexisting dimple clusters with varying sizes. These differently sized dimples were surrounded by tear ridges, reflecting the material’s fracture behavior under this temperature condition. As the microstructure in this region experienced only brief tempering effects, it showed no significant difference from the base metal’s microstructure, thereby demonstrating retained good toughness. This indicates that the material’s toughness was well preserved without notable brittle transition under 400 °C tempering conditions.

## 4. Discussion

### 4.1. Effect of Peak Temperature on HAZ Microstructure

**Figure 9:**
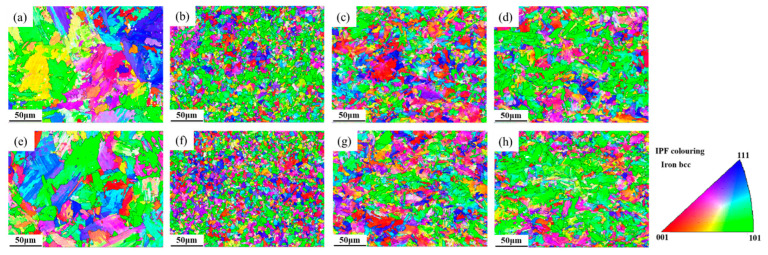
Inverse pole figures of test steel at different peak temperatures (**a**) Steel A 1350 °C; (**b**) Steel A 1000 °C; (**c**) Steel A 770 °C; (**d**) Steel A 400 °C; (**e**) Steel B 1350 °C; (**f**) Steel B 1000 °C; (**g**) Steel B 770 °C; (**h**) Steel B 400 °C.

**Figure 10:**
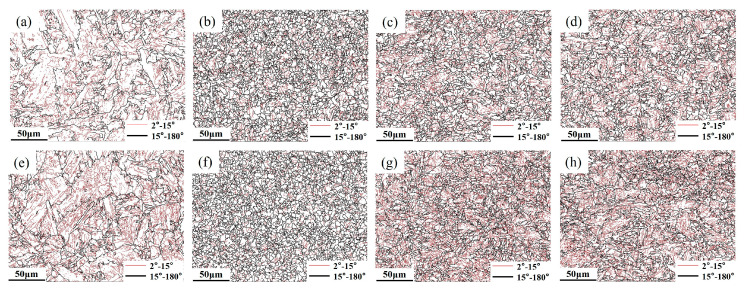
Grain boundary diagrams of experimental steel at different peak temperatures: (**a**) Steel A 1350 °C; (**b**) Steel A 1000 °C; (**c**) Steel A 770 °C; (**d**) Steel A 400 °C; (**e**) Steel B 1350 °C; (**f**) Steel B 1000 °C; (**g**) Steel B 770 °C; (**h**) Steel B 400 °C.

**Figure 11:**
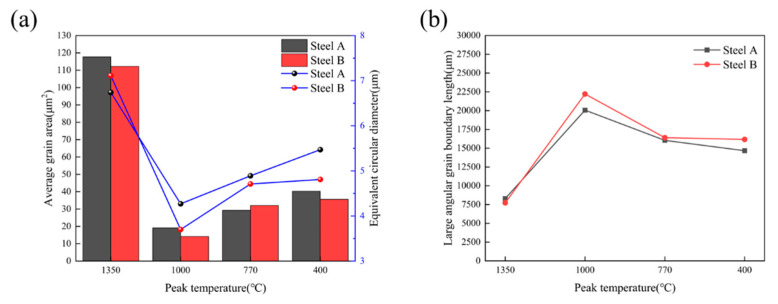
EBSD analysis of experimental steel at different peak temperatures: (**a**) grain size; (**b**) large angular grain boundary length.

Figure 9 and Figure 10 display the inverse pole figure (IPF) and low-angle/high-angle grain boundary distributions of the experimental steels under different thermal cycle peak temperatures. Statistical analysis was performed using Aztec Crystals software 2.1, with results shown in Figure 11. In EBSD analysis, grain size measurement serves as a critical parameter for evaluating material properties [33,34]. Figure 11a illustrates the grain size measurements for experimental steels with varying Cr contents, where both average grain area and equivalent circular diameter distribution are commonly employed to characterize grain dimensions. These parameters provide comprehensive insights into the material’s microstructure. By combining Figure 9 and Figure 11a, it can be visually observed that the experimental steels exhibit identical grain size evolution trends with decreasing peak temperatures. At 1350 °C, significant grain coarsening occurred in all experimental steels, with equivalent circular diameters showing an increasing trend as Cr content rose. The minimum grain size was achieved at a peak temperature of 1000 °C. When peak temperatures decreased to ICHAZ and SCHAZ ranges, grain sizes gradually increased until approaching the base metal’s dimensions. Previous studies suggest that chromium, as a strong carbide forming element, interacts with carbon to create fine, dispersed carbides along austenite grain boundaries [21]. These precipitates effectively inhibit austenite grain growth, resulting in a slight refinement of the grains in Steel B compared to Steel A with lower Cr content.

The effect of Cr content in experimental steels on the length of HAGB in the HAZ at different peak temperatures is shown in Figure 11b. It can be observed that the HAGB length in the experimental steels followed similar trends with varying peak temperatures, with minimal differences between the steels at the same temperature. The shortest HAGB length occurred at a peak temperature of 1350 °C. When the peak temperature decreased to 1000 °C, the HAGB length increased and reached its maximum value. As the peak temperature further dropped to 770 °C, the HAGB lengths of all two experimental steels returned to similar levels, measuring approximately 16,000 μm per unit area. At a peak temperature of 400 °C, the HAGB length showed a slight decrease compared to that at 770 °C, though not significantly. Studies have indicated that HAGBs effectively inhibit crack propagation, which explains why the experimental steels exhibited excellent low-temperature toughness at 1000 °C [35,36].

As can be seen from Figure 11, when the peak temperature is below 770 °C, the average grain size of Steel B shows no significant variation, and the length of high angle grain boundaries remains comparable. This indicates that the deterioration in impact toughness of Steel B at this stage is not closely related to the density of high-angle grain boundaries. Other contributing factors should be considered, particularly those involving secondary phases such as the quantity, morphology, and distribution of M-A constituent.

### 4.2. The Effect of M-A Constituent on Toughness in the ICHAZ

**Figure 12:**
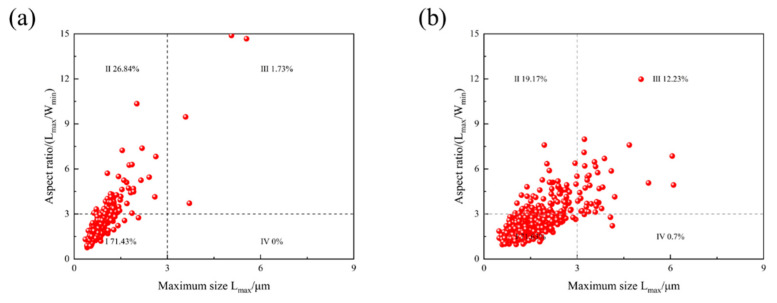
M-A component distribution of experimental steel ICHAZ: (**a**) Steel A; (**b**) Steel B.

From the impact toughness results, it is evident that Steel B exhibits overall low toughness in the ICHAZ, indicating the formation of a localized brittle zone. The morphology, volume fraction, and distribution of M-A constituent are the primary factors influencing the toughness degradation in the ICHAZ. To further investigate the effect of peak temperature on M-A constituent, SEM images of the experimental steel subjected to a peak temperature of 770 °C were analyzed. Table 2 summarizes the quantified density of M-A constituent per unit area. Based on their length and aspect ratio, M-A constituent was categorized into four types using a 3 μm threshold: dot-like, elongated, coarse elongated, and blocky (Figure 12) [37]. As shown in Figure 6, dot-like M-A constituent dominate in the ICHAZ of Steel B, accounting for over 50% of the total. Combined with Figure 12 and statistical data, increasing Cr content correlates with a rise in the number density of M-A constituent and a higher prevalence of elongated or angular M-A constituent. These elongated/angular M-A constituent are critically detrimental to toughness, as they act as stress concentrators and promote crack initiation [38]. The increased presence of such morphologies with Cr addition aligns with the observed deterioration in impact toughness.

**Figure 13:**
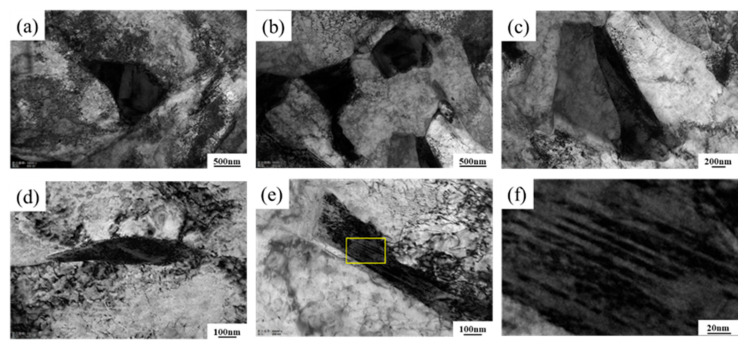
Microstructure of M-A components of Steel B in ICHAZ (770 °C): (**a**) TEM image of the sharp M-A components; (**b**) TEM images of block M-A components; (**c**) TEM images of rod M-A components; (**d**) TEM images of chain M-A components at grain boundaries; (**e**) TEM images of the long M-A block; (**f**) enlarged view of the yellow box in (**e**).

Further investigation into the structure and morphology of M-A constituent in Steel B at a peak temperature of 770 °C. Figure 12 illustrates the distribution of three distinct morphologies of M-A constituent in Steel B at 770 °C, sharp-tipped (pointed), blocky, and rod-shaped, all located near packet boundary regions. Microstructural analysis (Figure 13d) reveals that the grain boundary regions exhibit continuously distributed M-A constituent. The phase distribution pattern indicates that the martensite phase, characterized by a typical dislocation substructure (appearing as dark regions in the micrographs), is predominantly concentrated in the peripheral zones of the composite structure, while the retained austenite (light-colored regions) is localized in the core areas of the composite structure. Detailed characterization of the three M-A constituents demonstrates significant dislocation pile-up in their peripheral regions, particularly forming high-density dislocation networks at the tips of the composite structures. The local dislocation density in these regions markedly exceeds the inherent level of the bainitic matrix [39,40]. Notably, the presence of the martensite phase at the edges significantly alters the stress field distribution near phase boundaries, inducing a pronounced stress gradient at the interface. This abrupt mechanical state transition serves as a critical trigger for the initiation of interfacial microcracks [41]. Figure 13e,f present TEM images of the rod-shaped M-A constituent and a magnified view of the region marked by the yellow box, where micro-twins are observed. These micro-twins arise from the twinned martensitic transformation of high-carbon austenite. Compared to other types of M-A constituent, this morphology exhibits higher hardness, rendering it more susceptible to microcrack formation and subsequent material failure [42].

### 4.3. The Influence of Cr Element on the M-A Component of ICHAZ

**Figure 14:**
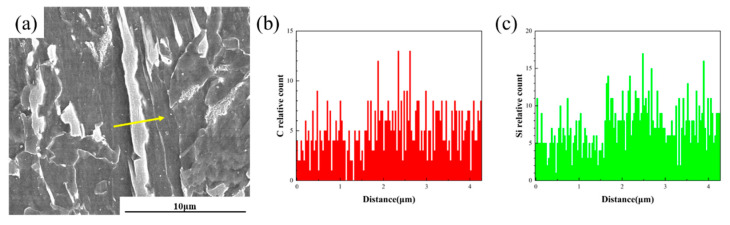
SEM and energy spectrum of chain M-A components at grain boundaries (Steel B, 770 °C): (**a**) SEM; (**b**) element C; (**c**) Si elements.

Figure 14 presents the scanning electron microscopy (SEM) image and corresponding energy-dispersive X-ray spectroscopy (EDS) analysis of chain-like M-A constituent formed along grain boundaries in Steel B after a thermal cycle with a peak temperature of 770 °C. A line-scan analysis was conducted to investigate the elemental distribution across the chain-like M-A constituent, with the scanning path (4.3 μm in length) illustrated in Figure 14a. Figure 14b,c reveal pronounced elemental enrichment, particularly in carbon (C) and silicon (Si), within the grain boundary-located M-A constituent. Notably, the C concentration exhibits a distinct peak signal at the M-A constituent regions, indicating significantly higher C content compared to the matrix. The formation of chain-like M-A constituent at grain boundaries is closely associated with localized C accumulation. As a strong carbide-forming element, Cr binds with carbon to form stable carbides (e.g., Cr_23_C_6_), reducing the free carbon concentration and diffusivity in austenite, thereby promoting heterogeneous austenitization in the ICHAZ [43]. During this process, undissolved carbides pinned the grain boundaries, creating carbon concentration gradients that led to localized C-rich microregions. Upon rapid cooling, these microregions preferentially transformed into core–shell M-A constituent, where the core consists of retained austenite with micro-twins, and the shell comprises high-hardness lath martensite. The synergistic effect of micro-twins and interfacial thermal mismatch stress induced localized stress concentration, triggering microcrack nucleation and subsequent toughness degradation. Furthermore, the prior austenite grain boundary regions inherently contain abundant vacancies and defects, which accelerate C diffusion and elevate C concentration near boundaries, thereby facilitating preferential precipitation of M-A constituent. Intriguingly, Si also shows moderate enrichment within the M-A constituent. These findings demonstrate that the formation mechanism of chain-like M-A constituent is governed by chemical heterogeneity and atomic diffusion kinetics, with such microstructural characteristics exerting profound impacts on material performance [14].

## 5. Conclusions

Under the premise of ensuring good resistance to S-CO_2_ corrosion of an experimental steel, the following conclusions were obtained through systematic investigation of the effect of Cr addition on HAZ microstructure and mechanical properties:

1. Addition of 0.5 wt.% Cr to the experimental steel reduced the −10 °C low temperature toughness of the base metal from 277 J to 235 J but improved its corrosion resistance in supercritical CO_2_ environments.
2. With 0.5 wt.% Cr addition to the experimental steel, embrittlement occurred in the ICHAZ subregion of the HAZ, where the low-temperature toughness decreased from 235 J of the base metal to 77 J. A peak in microhardness was also observed in this area, primarily due to partial austenitization during heating and subsequent formation of M-A constituent during rapid cooling.
3. As a strong carbide-forming element, Cr significantly reduces the carbon diffusion rate in austenite by forming stable carbides, inducing heterogeneous austenitization and localized carbon-enriched micro-zones in the ICHAZ. During rapid cooling, these carbon-rich regions preferentially transform into core–shell M-A constituent, characterized by retained austenite cores containing micro-twins and high-hardness lath martensite shells. The localized stress concentration arising from this microstructure synergizes with interfacial thermal mismatch stress, triggering microcrack nucleation and ultimately leading to material toughness degradation.

## Figures and Tables

**Table 1:** Chemical composition of X65 pipeline steel (wt.%).

Pipeline	C	Si	Mn	Cr	Ni	Mo	Cu	Nb	Ti
Steel A	0.05	0.2	1.31	0.21	0.2	0.11	0.1	0.039	0.011
Steel B	0.05	0.2	1.32	0.50	0.2	0.10	0.1	0.038	0.012

**Table 2:** Quantitative analysis of M-A constituent per unit area in experimental steels.

Experimental Steel Designation	Number of M-A Constituent/0.01 mm^2^
Steel A	231
Steel B	423

## Data Availability

The datasets presented in this article are not readily available because the data are part of an ongoing research project that has not yet been completed. Requests to access the datasets should be directed to 15105633440@163.com.

## References

[1] Niu A.J., Bi Z.Y., Wei F., Huang X.J., Liu B., Xi M.M. (2024). Research Progress and Development Trend of Pipes Used in New Energy Transportation in China. Welded Pipe Tube.

[2] Bi Z.Y., Huang X.H., Li Y.H., Wei F., Qin J.J., Wang L., Fan C.Q., Lu L. (2023). Development of HFW Steel Pipe for Supercritical Carbon Dioxide Transportation. Welded Pipe Tube.

[3] Zhao W.Q., Chen W.F., Yu C.L., Wang Z., Zhang J.S. (2023). Research Status of Carbon Dioxide Pipeline Transportation Technology. Pet. Chem. Equip..

[4] Tao Z., Du P., Wang X.Y., Hou S.Y. (2025). Bohai Equipment: Seizing the New Heights of CO_2_ Pipeline Transportation. China Pet. Petrochem..

[5] Zhao Y.Y., Chen X.W., Yang K., Wang B., Wang X.S. (2025). Development of X65 Steel Grade SAWL Pipe for CO_2_ Transportation. Welded Pipe Tube.

[6] Zhao Y.Y., Chen X.W., Li Y.P., Wang B., Wang X.S. (2024). Research Progress of CO_2_ Transmission Pipelines Technology. Welded Pipe Tube.

[7] Liu L.S. (2023). Study on corrosion characteristics of supercritical carbon dioxide pipeline. Chem. Eng..

[8] Gao Y.X., Pan J., Li Y., Zhang J., Li Y. (2024). Research Progress on the Corrosion of the Inner Surface of Pipeline Used for Transporting Supercritical Carbon Dioxide. Mater. Rep..

[9] Li K., Zeng Y., Luo J.L. (2021). Influence of H_2_S on the General Corrosion and Sulfide Stress Cracking of Pipelines Steels for Supercritical CO_2_ Transportation. Corros. Sci..

[10] Li K., Zeng Y. (2023). Long-term corrosion and stress corrosion cracking of X65 steel in H_2_O-saturated supercritical CO_2_ with SO_2_ and O_2_ impurities. Constr. Build. Mater..

[11] Zeng Y., Li K. (2019). Influence of SO_2_ on the Corrosion and Stress Corrosion Cracking Susceptibility of Supercritical CO_2_ Transportation Pipelines. Corros. Sci..

[12] Li K., Zeng Y. (2023). Advancing the mechanistic understanding of corrosion in supercritical CO_2_ with H_2_O and O_2_ impurities. Corros. Sci..

[13] Hu L.H., Zhang L., Xu L.N., Lu M.X., Chang W., Li Z.T. (2010). Effects of Cr content on microstructure and properties of low alloy corrosion resistant pipeline steel weld joints. Trans. Meter. Heat Treat..

[14] Li C. (2022). Effect of Carbon Equivalent on the Toughness of Coarse Grained HAZ of Girth Welded Joint of High-Grade Steel Pipeline. Ph.D. Thesis.

[15] Tang H.P. (2019). Study on Residual Stress and Microstructure Properties of Heat Affcted Zone During In-Service Welding on X80 Natural Gas Pipeline. Ph.D. Thesis.

[16] Wu Z.W., Dai Z.J., Wang L.L., Zhang P.C., Wu H.B. (2025). Development of X65 Pipeline for Supercritical CO_2_ Transport Environment. Steel Pipe.

[17] Wang B. (2019). Study of Corrosion and Erosion Mechanism for Cr-Bearing Alloy Steel in CO_2_-Containing Wet Gas Pipeline. Ph.D. Thesis.

[18] Qiao S., Yu J.S., Wang H.L., Zhou X.G. (2022). Research Progress on Corrosion Behavior of Supercritical Carbon Dioxide in Fourth Generation Nuclear Reactors. Mater. Res. Appl..

[19] Wang C.L., Guo H.D., Fang J., Yu S.X., Yue X.Q., Hu Q.H., Liu C.W., Zhang J.W., Zhang R., Xu X.S. (2023). The role of Cr content on the corrosion resistance of carbon steel and low-Cr steels in the CO_2_-saturated brine. Pet. Sci..

[20] Lee H.J., Lee H.W. (2015). Effect of Cr Content on Microstructure and Mechanical Properties of Low Carbon Steel Welds. Int. J. Electrochem. Sci..

[21] Li H.C., Chen S.Y., Yue X.D., Yang H.M. (2014). Effect of Cr on microstructure and austenite-ferrite transformation temperature of low carton steel. Heat Treat. Met..

[22] Heo N.H., Heo Y., Kwon S.K., Kim N.J., Kim S.J., Lee H.C. (2018). Extended Hall–Petch Relationships for Yield, Cleavage and Intergranular Fracture Strengths of bcc Steel and Its Deformation and Fracture Behaviors. Met. Mater. Int..

[23] Hong R., Liu H.C., Zhu X.D., Liu N.S., Yin S.B., Ma Q.L., Jia S.J. (2024). Effect of Cr Element in Gas-Shielded Solid Wire for Oil and Gas Long-Distance Pipeline on Microstructure and Low Temperature Toughness of Weld. Materials.

[24] Wang F., Fan Y.R., Zhang X.X., Sun Q.F. (2014). Effect of Cr in Self-shielded Flux-cored Wires on Sharpy Impact Performance and Microstructure of the Weld Metals. Han Guan.

[25] Hou Y. (2023). Effect of alloying element on microstructure and properties of girth welded joints of X80 pipeline steel. Metall. Anal..

[26] Wang Y.F. (2021). Effect of Chromium on Rust Layer Structure, Microstructure and Properties of Weathering Bridge Steel. Ph.D. Thesis.

[27] (2016). Carbon and Low-Alloy Steel—Determination of Multielement Contents—Spark Discharge Atomic Emission Spectrometric Method (Routine Method).

[28] Li C. (2017). Welding Procedure Study of Corrosin Resistant Steel for Ships. Ph.D. Thesis.

[29] (2020). Metallic materials—Charpy pendulum impact test method.

[30] Tang C.J., An T.B., Peng Y., Li C.C., Ma C.Y., Liu X.M. (2024). Effect of heat input on microstructure and mechanical properties of weld metal of 690 MPa grade HSLA steel. Trans. China Weld. Inst..

[31] Zhou X.Q. (2023). Microstructure Evolution and Impact Properties of Low Carbon Bainitic Steels. Ph.D. Thesis.

[32] Wang X.Y., Li L., Wu Q.D., Wang C.C., Liang X.D., Yang P., Wang X. (2021). Effect of austenitizing temperature on microstructure and mechanical properties of rolled C61 steel. Trans. Mater. Heat Treat..

[33] Di Y.J., Ping L.L., Tang X.C., Zhang Z.J., Cheng G.H. (2025). Effect of annealing temperatures on non-magnetic structural steel 20Mn23AlV. J. Iron Steel Res..

[34] Liu H., Luo R., Qian X.G., Sun X., Zhao Q.L., Cui L.Y., Yang C., Ding H.N. (2024). Effect of Heat Treatment Process on Properties and Microstructure of UNS N07718 Superalloy for Deep-sea Oil and Gas Development. J. Netshape Form. Eng..

[35] Cao S.L., Zhang Q.J., Zhang C.J. (2017). Influence of linear energy on microstructure and properties of heat affected zone of EH420 offshore steel. Chian Metall..

[36] Yu Y., Gao Z.Y., Gong B.M. (2025). Fracture toughness of CGHAZ of X80 pipeline steel in hydrogen sulfide environment. Weld. Join..

[37] Lan L., Qiu C., Zhao D., Gao X. (2011). Microstructural characteristics and toughness of the simulated coarse-grained heat affected zone of high strength low carbon bainitic steel. Mater. Sci. Eng. A.

[38] Matsuda F., LI Z., Bernazovsky P., Ishihara K., Okada H. (1991). An investigation on the behaviour of the M/A constituent is simulated HAZ of HSLA steels. Weld World.

[39] Duan H. (2022). Study on Microstructure Control and Strengthening & Toughening Mechanism of High Strength Pipeline Steel for Low Temperature Application. Ph.D. Thesis.

[40] Huang K.L., Zhao X., Zuo X.R., Wang H.H. (2020). Study on Low Temperature Fracture Behavior of X80 Pipeline Steel with Multiphase Microstructure. Hot Work. Technol..

[41] Li Y. (2023). Effect of Mo on the Toughness of HAZ of Girth Welded Joint of X80 Pipeline Steel. Ph.D. Thesis.

[42] Li B., Zhou X., Jia S.J., Chen X.P., Fu S., Zhao D.L., Zhang H.A., Guo J. (2024). Study on Fracture Behavior and Toughening Mechanisms of Ultra-High-Strength Pipeline Steel. Metals.

[43] He X.M., Chen R.W. (1986). An Investigation on the Diffusion Coefficient of Carbon in Austenite. Met. Sci. Technol..

